# Synaptic FUS Localization During Motoneuron Development and Its Accumulation in Human ALS Synapses

**DOI:** 10.3389/fncel.2019.00256

**Published:** 2019-06-12

**Authors:** Dhruva Deshpande, Julia Higelin, Michael Schoen, Thomas Vomhof, Tobias M. Boeckers, Maria Demestre, Jens Michaelis

**Affiliations:** ^1^Institute of Biophysics, Ulm University, Ulm, Germany; ^2^Institute for Anatomy and Cell Biology, Ulm University, Ulm, Germany; ^3^German Center for Neurodegenerative Diseases (DZNE), Ulm Site, Ulm, Germany

**Keywords:** FUS, ALS, hiPSCs, motoneuron synapses, super-resolution microscopy, SMLM

## Abstract

Mutations in the fused in Sarcoma (*FUS*) gene induce cytoplasmic FUS aggregations, contributing to the neurodegenerative disease amyotrophic lateral sclerosis (ALS) in certain cases. While FUS is mainly a nuclear protein involved in transcriptional processes with limited cytoplasmic functions, it shows an additional somatodendritic localization in neurons. In this study we analyzed the localization of FUS in motoneuron synapses, these being the most affected neurons in ALS, using super-resolution microscopy to distinguish between the pre- and postsynaptic compartments. We report a maturation-based variation of FUS localization in rodent synapses where a predominantly postsynaptic FUS was observed in the early stages of synaptic development, while in mature synapses the protein was entirely localized in the axonal terminal. Likewise, we also show that at the synapse of human motoneurons derived from induced pluripotent stem cells of a healthy control, FUS is mainly postsynaptic in the early developmental stages. In motoneurons derived from ALS patients harboring a very aggressive juvenile FUS mutation, increased synaptic accumulation of mutated FUS was observed. Moreover increased aggregation of other synaptic proteins Bassoon and Homer1 was also detected in these abnormal synapses. Having demonstrated changes in the FUS localization during synaptogenesis, a role of synaptic FUS in both dendritic and axonal cellular compartments is probable, and we propose a gain-of-toxic function due to the synaptic aggregation of mutant FUS in ALS.

## Introduction

Fused in Sarcoma is a highly conserved nucleic acid-binding protein predominately localized in the cellular nucleus (Ling, 2018). The human *FUS* gene codes for a 526 amino acid protein that can be divided into an N-terminal end consisting of a prion-like stretch or low complexity domain enriched in glutamine, glycine, serine, and tyrosine residues (Q/G/S/Y), followed by a glycine-rich area. The C-terminal portion contains an RNA recognition motif, an arginine-/glycine-rich domain, followed by a zinc-finger motif, and a nuclear localization signal (NLS) at the end (Schwartz et al., 2015; Shang and Huang, 2016). FUS shows binding to both single- and double-stranded DNA as well as RNA, and is involved in several cellular functions including DNA damage repair (Qiu et al., 2014) and transcription regulation (Fujioka et al., 2013). Further, it is also known to shuttle between the nucleus and cytoplasm, where it has a role in mRNA transport and translation (Ishigaki and Sobue, 2018). In neurons, FUS shows an additional localization in neurites, where it has been detected in ribonucleoprotein complexes (RNPs) (Belly et al., 2005; Khalil et al., 2018). FUS plays a role in dendritic maturation and spinogenesis (Fujii and Takumi, 2005), while in the axon it is involved in microtubule growth and organization (Yasuda and Mili, 2016), and is known to control the expression of axonal transport associated proteins (Groen et al., 2013). FUS is further described to be localized at synapses, as demonstrated in cortical and hippocampal neurons (Fujii et al., 2005; Schoen et al., 2016), where it is reported to have a role in synaptic functions especially in regulation of proteins involved in synaptic transmission (Udagawa et al., 2015; Sproviero et al., 2018). At the dendritic spine FUS is known to influence the local protein synthesis machinery and hence it is involved in controlling the synaptic plasticity (Qiu et al., 2014; Sephton and Yu, 2015).

The role of FUS in neurons recently gained importance when, along with a related RNA-binding protein TDP-43, it was found to be involved in the neurodegenerative disorders – ALS and frontal temporal dementia (FTD) (Arai et al., 2006; Neumann et al., 2006, 2009; Kwiatkowski et al., 2009; Vance et al., 2009). While in ALS a gradual degeneration of upper and lower motoneurons followed by atrophy of the connected muscles is observed, FTD shows a progressive neuronal loss across the frontal and temporal cortices (Mackenzie et al., 2010). Both diseases are characterized by the presence of abnormal protein aggregates, of which FUS is found to be the main component in certain disease subtypes. However, while a mutated FUS (mFUS) protein is generally identified in ALS pathologies, most FTD cases reported so far show accumulation of only wild-type FUS with no linked mutations (Ravanidis et al., 2018). About 1% of sporadic and 4% of familial ALS cases can be attributed to the mutation of the *FUS* gene and the subsequent protein mislocalization in the affected motoneurons (Ling et al., 2013; Renton et al., 2014). The ALS-linked mutations in FUS mostly occur in either the low-complexity domain of the protein or in the nuclear localization sequence region, both of which can cause a cytoplasmic accumulation of FUS (Shang and Huang, 2016). mFUS is also found to cluster in stress granules localized in the cytoplasm and along neurites (Yasuda et al., 2013; Higelin et al., 2016). The synapses are also affected, with reduced dendritic spine numbers reported in the cortical and motoneurons of mutant FUS rodent models (Sephton et al., 2014; Herms and Dorostkar, 2016). Many microRNAs and proteins involved in synaptic functions have also been found to be deregulated by mFUS (Udagawa et al., 2015; De Santis et al., 2017; Yokoi et al., 2017).

As FUS plays an important role in synapse formation and regulation, and its mutations affect these functions, it is important to know the precise distribution and localization of FUS in synapses. In our recent published work we have shown a strong FUS expression throughout the adult rat brain and specifically localized it in the presynaptic compartments of rat hippocampal neurons (Schoen et al., 2016). However, as FUS aggregation in the hippocampal area is an FTD characteristic, while in ALS pathology a similar accumulation is seen in motoneurons (Nolan et al., 2016), it is thus relevant in an ALS context, to study FUS localization specifically in healthy motoneurons and compare it to those harboring a FUS mutation. In this study, we used SR optical microscopy to determine the exact localization of FUS within the synaptic compartment. The distances in the pre- and postsynaptic compartments are smaller than the diffraction limit making it impossible to distinguish between them with a normal optical microscopy (Heller and Rusakov, 2017), however, with SR microscopy it is possible to observe such small distances with high resolution (Betzig et al., 2006; Hell, 2007; Sauer and Heilemann, 2017; Sahl et al., 2017). The position of individual fluorophores is determined with high precision in the SR technique of SMLM, leading to a super-resolved image of labeled bimolecular structures (Rust et al., 2006; Heilemann et al., 2008; Jungmann et al., 2014). Importantly, SMLM has been applied previously in neurons for synaptic imaging (Dani et al., 2010; Ladepeche et al., 2013; Andreska et al., 2014; Wang et al., 2017).

Using SMLM to image rat and hiPSCs derived motoneurons, we have shown here that FUS localizes predominantly in the dendritic compartment close to the postsynaptic marker Homer1 during development, while in more mature synapses it clusters closer to Bassoon in the presynaptic bouton. Furthermore, we observed an increased density of FUS clusters in the neurites of hiPSCs derived motoneurons from an ALS patient carrying FUS mutation, as compared to control cells. Interestingly, mFUS in patient neurons strongly aggregated at synapses along with an increased accumulation of pre- and postsynaptic proteins Bassoon and Homer1.

## Materials and Methods

### Human and Animal Ethics Statement

All procedures with human material were in accordance with the ethical committee of Ulm University (Nr.0148/2009 or 265/12). The use of human material was approved by the Declaration of Helsinki concerning Ethical Principles for Medical Research Involving Human Subjects (Stockmann et al., 2013a). All participants gave informed consent for the study.

All animal experiments were performed in compliance with the guidelines for the welfare of experimental animals issued by the Federal Government of Germany, the National Institute of Health, and the Max Planck Society. The experiments in this study were approved by the review board of the Land Baden-Württemberg (Permit Number O.103).

### Cell Culturing

#### Reprogramming of Human Keratinocytes and Cultivation of hiPSCs

Generation of human iPSC cells from reprogrammed human keratinocytes was performed as previously described, using a lentiviral multicistronic vector (Takahashi and Yamanaka, 2006; Aasen et al., 2008). Two previously described cell lines from a healthy volunteer and an ALS patient with an Asp502Thrfs^∗^27 FUS mutation were generated (Stockmann et al., 2013a; Higelin et al., 2016). Briefly, plucked human hair samples were cultivated in conditioned mouse embryonic fibroblast medium supplemented with 50 μg/ml ascorbic acid (Sigma–Aldrich, Germany), 10 ng/ml fibroblast growth factor-FGF2 (Cell Guidance Systems), and 10 μM Rho-associated kinase (ROCK) inhibitor (Ascent Scientific). After outgrow of keratinocytes from the hair root, medium was replaced to Epilife (Gibco, M-EPICF, Big Cabin, OK, United States) supplemented with 10 μM ROCK inhibitor. To achieve efficient reprogramming cells were not passaged more than two times with k-dispase (BD Bioscience). Viral infection was performed using polybrene (Sigma–Aldrich, Germany) on two consecutive days. Subsequently, cells were seeded on irradiated rat embryonic feeder cells and cultivated in hiPSC medium [knockout DMEM (Gibco, Big Cabin, OK, United States), 20% knockout serum replacement (Thermo Fisher Scientific), 1% non-essential amino acids (NEAAs, Gibco, Big Cabin, OK, United States), 1% GlutaMax^TM^, 100 μM β-mercaptoethanol (Millipore), 1% Antibiotic-Antimycotic (Thermo Fisher Scientific) supplemented with 50 μg/ml ascorbic acid, 10 ng/ml FGF2, and 10 μM ROCK inhibitor]. Colonies showing iPSC morphology were mechanically lifted and cultivated under feeder-free conditions with mTeSR1 medium (Stem Cell Technologies) at 37°C, 5% CO_2_. HiPSCs were kept in culture for not more than a maximum of 35 passages. Cell lines were characterized by performing pluripotency tests using the StemLite Pluripotency Kit (Cell Signaling), to check for pluripotency markers and the spontaneous germ layer differentiation test as previously published (Linta et al., 2012; Stockmann et al., 2013a). Karyograms of generated hiPSC lines were analyzed for chromosomal aberrations induced by reprogramming. Shortly, mitosis was inhibited via incubation with 0.6 mg/ml colchicine (Eurobio) in mTeSR1 for 2 h at 37°C. Cells were detached with TrypLE (Invitrogen, Germany) for 4 min at 37°C and analyzed in 3 ml of mTeSR1. Analysis was kindly done by the Institute for Human Genetics, Ulm University Hospital.

Further differentiation of hiPSCs into motoneurons was performed according to published protocols (Hu and Zhang, 2009; Stockmann et al., 2013a). For embryoid bodies (EBs) formation, hiPSCs were cultured in suspension in EB medium [DMEM/F12 (Gibco, Big Cabin, OK, United States), 20% knockout serum replacement, 1% NEAAs, 1% β-mercaptoethanol, 1% Antibiotic-Antimycotic] in ultra-low attachment flasks (Corning Costar) supplemented with 10 μM ROCK inhibitor for the first 24 h. Neuronal differentiation induction was induced on day 4 by replacing with differentiation medium containing DMEM/F12, 1% NEAA, 2 μg/ml heparin (Sigma–Aldrich, Germany), 1% Antibiotic-Antimycotic, 2% hormone mix [24 nM sodium selenite + 16 nM progesterone + 0.08 mg/ml apotransferrin + 7.72 μg/ml putrescin (all Sigma–Aldrich, Germany) + 0.02 mg/ml insulin (SAFC)] supplemented with 10 ng/ml glial derived neurotrophic factor (GDNF), 10 ng/ml brain derived neurotrophic factor (BDNF), 10 ng/ml insulin growth factor (IGF-1) (all Peprotech), 0.1 μM cAMP (Sigma–Aldrich, Germany), and 20 μg/ml ascorbic acid. On day 7, EBs were seeded in differentiation medium on laminin (Sigma–Aldrich, Germany)-coated plates and then 0.1 μM of retinoid acid (Sigma–Aldrich, Germany) was added on day 10. On day 15, neurospheres were transferred into T75 low-attachment flasks and further cultivated in the same medium with addition of 1 μM purmorphamine (Calbiochem), 0.1 μM retinoid acid, and 2% B27 (Gibco, 12587, Big Cabin, OK, United States). At day 28, neurospheres were seeded on coverslips pre-coated with poly-L-ornithine (Sigma–Aldrich, P4957, Germany) and laminin. Cells were cultivated in differentiation medium plus 0.05 μM retinoid acid, 0.5 μM purmorphamine, and 2% B27 further on. After 42 days (DIV 42) of cultivation cells were fixed and used for further experiments.

#### Rat Motoneuron Culture

The rat embryonic primary motoneurons culture technique was adapted from previous publications (Martinou et al., 1989; Stockmann et al., 2013b). In brief, 15.5 days pregnant Sprague–Dawley rats were anesthetized with 100% CO_2_ and then killed by decapitation. Embryos were extracted from the uterus and then placed on ice in a buffer of Hanks’ balanced salt solution (HBSS) supplemented with 1% penicillin–streptomycin and 1% glucose (all from Invitrogen, Germany). The embryos were then decapitated and the spinal cord harvested with careful separation from the surrounding sheath and cut in small pieces and shortly washed in dulbecco’s phosphate-buffered saline (DPBS). The tissue was digested by trypsinization for 15 min followed by incubation with DNase 1 (40 mg/ml, Sigma–Aldrich, Germany) for 10 min at 37°C. Cells were then teased apart by gentle titration with a fire-smoothed Pasteur pipette and the obtained single cell solution carefully loaded on top of cell media bed (32% DMEM, 64% knockout-DMEM/F12, 2% B27 supplement, 1% penicillin/streptomycin, and 1% NEAA; all from Gibco, Big Cabin, OK, United States) with 3.5% bovine serum albumin (BSA) and centrifuged at 80 ×*g* for 20 min at room temperature (RT). The resultant pellet was resuspended in DPBS and loaded on a Nycodenz (Serva, Germany) gradient, consisting of three distinct density layers, followed by centrifugation for 20 min with 700 ×*g* at 4°C. The upper layer, containing mostly motoneuronal cells, was collected and centrifuged at 1000 ×*g* for 10 min at 4°C. About 10^5^ of the obtained cells were plated for attachment on poly-L-ornithine and laminin-coated glass coverslips for incubation with cell medium supplemented with 1% knock-out serum replacement at 37°C in a 5% CO_2_ environment. After 2 h the medium was replaced to one without knock-out serum, and cells were incubated for up to 14 or 21 days.

#### Adult Mice Spine Sections

Spinal cord samples were obtained as previously reported (Saxena et al., 2013). Briefly, mice were perfused with cold PBS followed by 4% paraformaldehyde (PFA), spinal cords were carefully dissected and post fixed in 4% PFA for 18 h at 4°C, cryoprotected in 30% sucrose, embedded in OCT (TissueTek), and sectioned at 30 μm at the lumbar part region using a vibratome. Free-floating sections were then used for immunohistochemistry.

### Crude Synaptosomal Fractionation

Subcellular fractionation of spinal cords (*n* = 17) or cells (5–15 dishes) was performed as previously described with minor modifications (Distler et al., 2014; Reim et al., 2017). All steps were performed at 4°C. Spinal cords from mice were dissected and homogenized in 0.32 M sucrose, 5 mM hydroxyethyl-piperazineethane-sulfonic acid (HEPES), pH 7.4, and centrifuged at 1000 ×*g*. Spinal cord homogenates or cells that were previously homogenized in PBS were centrifuged at 12,000 ×*g* for 15 min. The pellet (P2) was incubated with 1 mM Tris–HCl on ice and centrifuged at 32,000 ×*g*. This fraction (S2) was then solubilized in a 0.32 M sucrose, 5 mM Tris–HCl, pH 8.1 buffer, and loaded on a discontinuous sucrose step gradient (0.85 M/1.0 M/1.2 M). After centrifugation at 85,000 ×*g*, the synaptosomes (Sy) were collected from the 1.0 M/1.2 M interphase. Equal amounts of 10 μg from each sample were separated via SDS–PAGE and subsequently blotted on Nitrocellulose membranes according to standard protocols. Incubation with the primary antibody was followed by incubation with an horseradish peroxidase (HRP)-conjugated secondary antibody (Dako). Signals were visualized with the Pierce ECL Western Blotting Substrate and further detected with the MicroChemi 4.2 machine.

### Immunocytochemistry

Cells grown on coverslip were fixed with either 4% PFA (EM-grade, VWR, Germany) at RT for 20 min or with cold methanol at -20°C for 10 min, followed by three washing steps with PBS for 5 min. Fixed cells were then permeabilized and blocked for 2 h at RT with PBS blocking buffer containing 0.3% Triton-X, 10% goat serum, and 5% fetal bovine serum (FBS). Thereafter, primary antibodies, diluted in 1:10 of blocking buffer, were incubated with cells overnight at 4°C, after which excess antibodies were subsequently removed by washing with PBS for three times for 30 min each. Dye labeled secondary antibodies were also diluted similarly and incubated for 2 h at RT followed by PBS washing for three times with 10 min for each. Cells were then post-fixed and washed similar to their pre-fixation method and either imaged immediately or stored in PBS at 4°C for a maximum duration of 1 week. Free-floating tissue sections from murine spine were incubated in a solution of 0.3% Triton-X with 3% BSA in PBS for 2 h at RT, for permeabilization and blocking. Primary antibodies were applied for 48 h at 4°C and the secondary antibody for 2 h at RT both diluted in the blocking buffer. Both incubation steps were followed, respectively, by three PBS washes at RT for 30 min.

For confocal microscopy, cells/tissue sections were immediately mounted and sealed with ProLong Gold Antifade reagent with DAPI (Invitrogen). During imaging, motoneuron cells were identified as those labeled by NF-H antibody.

Rabbit anti-FUS (Sigma–Aldrich, Germany, Cat# HPA008784, RRID:AB\_1849181), mouse anti-PSD95 (Abcam, Cat# ab2723, RRID:AB\_303248), guinea pig anti-Homer1 (Synaptic Systems, Cat# 160 004), and guinea-pig anti-MAP2 (Synaptic Systems, Cat# 188 004, RRID:AB\_2138181) primary antibodies were diluted 1:800. Mouse anti-Bassoon against N-terminus (Enzo Life Sciences, Cat# ADI-VAM-PS003, RRID:AB\_10618753), rabbit anti-Bassoon against the C-terminus (Synaptic Systems, Cat# 141 003, RRID:AB\_887697), and guinea-pig anti-VAChT (Synaptic Systems, Cat# 139 105 RRID:AB\_10893979) were diluted 1:500. The chicken anti-NF-H chain (Abcam, Cat# ab4680, RRID:AB\_304560) primary antibody was diluted 1:3000. An alternative rabbit anti-FUS antibody (Bethyl Cat# A300-302A, RRID:AB\_309445) was used at a dilution of 1:800.

The secondary antibodies (all from Life Technologies) had either Alexa Fluor^®^647 (red-channel) or 532 (green-channel) dye when used for SMLM, while for confocal microscopy Alexa Fluor^®^405, 488, 564, and 647 dyes were used. For a primary antibody dilution of 1:500, 1:800, or 1:3000, the secondary antibodies were diluted 1:800, 1:1000, or 1:5000, respectively.

### Confocal Imaging

All confocal images were obtained with a commercial Leica DMi8 inverted microscope using either 40× or 60× oil-immersion objective. The images were acquired in the LAS X software and further image processing was done in Image J (v 1.52c; National Institutes of Health).

### Electron Microscope Imaging

Human iPSC-derived motoneurons were grown on sapphire discs. Later the discs were clamped between two aluminum planchettes in a 100-μm deep cavity. Samples were high pressure frozen using a Wohlwend HPF Compact 01 high-pressure freezer (Engineering Office M. Wohlwend GmbH). Freeze substitution was performed as described (Halbedl et al., 2016). The substitution medium consisted of acetone with 0.2% osmium tetroxide (Plano Agar), 0.1% uranyl acetate (Merck), and 5% of water. After substitution for 18 h from -90°C to RT, samples were washed with acetone (Sigma) and gradually embedded in Epon (Fluka). Ultra-thin sections (75–80 nm) were cut parallel to the sapphire disc with a Leica Ultracut UCT ultramicrotome using a diamond knife (Diatome). This was kindly done by the central EM unit (Ulm University). Samples were imaged with a JEOL 1400 Transmission Electron Microscope (JEOL) and the images were digitally recorded with a Veleta camera (Olympus).

### SMLM Imaging

Single molecule measurements were performed on a custom built wide-field setup. Continuous-wave lasers of 405, 647 (Toptica Photonics, Germany), and 532 nm (Cobolt, Sweden) were used for activation and excitation. The laser lines were combined in an Acousto-optical tunable filter (AOTF; Gooch & Housego, United Kingdom), passed through a clean-up filter (AHF, Germany), and focused on the back focal plane of an oil immersion objective (60× APO TIRF, NA 1.49 Oil, Nikon) to create an oblique illumination. The fluorescence emission was separated first from the excitation light with dichroic mirrors and later into two different detection channels by combination of dichroic mirrors and emission filters (all from AHF Analysentechnik, Germany), which were then measured on separate EMCCD cameras (iXon 897 and iXon Ultra 897 Andor Technology, United Kingdom). The z-drift of the samples was corrected with a home built auto-focus system (Hellen and Axelrod, 1990). The samples used for SMLM were always cultured on a high precision glass coverslip of 170 μm thickness (Carl Roth).

#### dSTORM Technique

The method of dSTORM was used for obtaining two-color sub-diffraction resolution images. For imaging, the samples were placed in a special oxygen scavenger buffer with a pH of 7.4 containing 100 U/ml glucose oxidase, freshly prepared 100 mM cysteamine, 400 U/ml of catalase, and 40 mg/ml of glucose (all Sigma–Aldrich, Germany) mixed in degassed PBS. For both channels, a series of 30,000 images were recorded at 20 ms exposure time, with the red channel (Alexa Fluor^®^647) measured first followed by the green (Alexa Fluor^®^532).

#### Exchange-PAINT Technique

Material preparation and DNA-points accumulation for imaging in nanoscale topography (DNA-PAINT) techniques described in Schnitzbauer et al. (2017) were adapted for this study. For SR color multiplexing, samples were immunolabeled with primary antibody as described in the section “Immunocytochemistry.” Secondary antibodies against guinea-pig and mouse were tagged with different DNA docking strands and their respective complimentary sequences, labeled with Cy3B dye, were used as imager strands. The DNA sequence P6 (5′-TTTTAGGTAAA-3′) and P9 (5′-TTAATTAGGAT-3′) were used, respectively, for mouse and guinea-pig antibodies (Schnitzbauer et al., 2017). In order to choose FUS-positive synapses, FUS was labeled with a normal anti-rabbit secondary antibody with Alexa Fluor^®^647 and imaged with the above mentioned dSTORM technique (the section “dSTORM Technique”). Before measurement samples were incubated with gold beads (80 nm gold beads, BBI solutions, United Kingdom) for tracking the drift during imaging. First the SR images of the FUS protein in the red channel were obtained. The oxygen scavenger buffer was exchanged with an imager buffer (PBS with 500 mM NaCl, pH 7.2) containing the P6 imager strand and then 40,000 images were measured at 200 ms exposure time. The P6 imager strand was washed out with washing buffer (PBS, pH 7.2) till no trace of fluorescence was visible. The third color was measured in a similar manner with P9 imager strand.

#### SMLM Data Processing

All the single molecule data reconstruction was done with the MATLAB-based FIRESTORM software as previously described (Schoen et al., 2016). Briefly, the center of every single molecule localization was determined by their COM and several such signals were filtered based on intensity, asymmetry, and width thresholds. The selected localizations were reconstructed in a 10 nm pixel raster weighted by their intensity. Green channel images were then adjusted to the red channel based on a transformation function obtained from fluorescent beads imaged in both channels (100 nm four-color labeled beads, TetraSpek Microspheres, Life Technologies). For the dSTORM images, the *xy*-drift was corrected in the post-processing with a redundant cross correlation algorithm implemented in the FIRESTORM software (Wang et al., 2014). A drift correction function based on bead-tracking was used for the DNA-PAINT measurements.

### Image Analysis

All the further image analysis was done in ImageJ. Unless indicated otherwise, FUS is always depicted in red color, while markers Bassoon, Homer1, and PSD95 are in green, cyan, and magenta, respectively, in all the SMLM images. The reconstructed images were used for area and distance analysis without any further processing. The images depicted in the figures are processed for brightness/contrast and then displayed with a 0.7 Gaussian blur.

#### Distance Analysis for Synaptic Proteins

Colocalizing protein clusters in two-color SMLM images were selected manually as individual synapses. The synapses were marked with a rectangle having 400 nm as its longer side (orientated along the trans-synaptic axis). This cut-off distance of 400 nm was based on the average size of synapses previously determined by SR and EM (Dani et al., 2010; Stockmann et al., 2013a). The COM of each cluster was determined along both *x*- and *y*-axis. The Euclidean distance within the two synaptic proteins was then calculated as the difference between their respective COM. Distances of several synapses were thus calculated and grouped in a histogram with 10 nm binning. The mean of top 40% of all the measured distances, rounded up to the nearest tenth, was considered as the average distance between the two protein clusters. One should note that while for synapses with an overall extension exceeding the spatial resolution of the SR microscopy approach, the localization error is the main contributing error, for the case of maturing synapses with dimensions not much larger than the resolution limit, also the size of the synapse itself factors into the experimental. As a result, the reported values for maturing synapses have an additional error. Images from more than five biological replicates were used for the distance analysis.

#### Distinguishing Smaller and Larger Synapses in Rat Motoneurons

The size of a synapse was determined by the area of the protein clusters (Supplementary Figure 7C). All colocalizing protein clusters <0.03 μm^2^ were considered as smaller (developing) synapses in rat primary motoneuronal cultures, to distinguish them from larger (mature) synapses (>0.03 μm^2^).

#### Cluster Area Analysis

Open-source software SR-Tesseler was used for analysis of protein cluster area in the SMLM images (Levet et al., 2015). In this software first, the area among individual localizations is computed using Voronoï tessellation. Next, objects in the image (in our case the neurites) are identified by setting a density threshold which is automatically determined by the program based on the average number of localizations within the image area. In our analysis, the density threshold was determined to be twice the average localization density, similar to an ideal situation described in Levet et al. (2015). Finally, clusters (in our case protein clusters) were identified within the defined objects and their area was then determined by setting a density threshold of 3 and a minimal area of 0.0005 μm^2^. For the analysis of protein cluster areas within the synapses, a region of interest (ROI) was defined around a synapse previously identified in the SMLM image (as described in the section “Distance Analysis for Synaptic Proteins”). The cluster identification step in the SR-Tesseler was then computed within the ROI. For synaptic cluster analysis, all the synapses in images obtained from three separate cell cultures (biological replicates *n* = 3) were analyzed.

#### Statistical Analysis

All statistical analysis was done with GraphPad Prism version 7.4 (GraphPad Software Inc., La Jolla, CA, United States) program. The statistical significance of two data-groups was analyzed with a two-tailed unpaired *t*-test. The significance for more than two data groups was tested with a one-way ANOVA test and Tukey’s multiple comparison test. Results are represented as approximate mean values ± standard error of the mean (SEM). Statistical significance levels were set to *p* ≤ 0.05. An outlier analysis using the ROUT method with a *Q* = 0.5% was conducted for synaptic cluster analysis data. The identified outliers were removed in the subsequently plotted graph, however, no change in the statistical significance was observed (Figure 6E and Supplementary Figure 7C).

## Results

### FUS Distribution in hiPSC Derived Control Motoneurons

**FIGURE 1:**
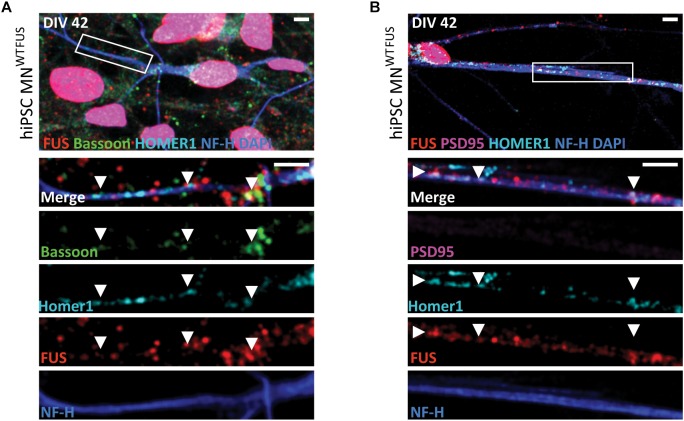
Subcellular location of FUS in control hiPSC derived motoneurons. Confocal images of human MN^WTFUS^ cells, identified by the NF-H (blue) labeled axon, show immunolabeled FUS (red) in the nucleus coinciding with DAPI (blue). The first row depicts an overview image with the magnified crop (white box) shown as merged or single channel images in the lower rows. **(A)** FUS is present along the neurites overlapping with pre-synaptic marker Bassoon (green) and postsynaptic marker Homer1 (cyan) indicated by the white arrowheads. **(B)** Cells at this culture stage (DIV 42) showed no presence of PSD95 (magenta) in FUS (red) and Homer1 (cyan)-positive punctae (white arrowheads). All scale bars 5 μm.

In order to examine FUS localization in human motoneurons, we used cultures derived from hiPSC cell lines established from a healthy volunteer (hereafter abbreviated as human MN^WTFUS^). This cell line has already been extensively characterized previously (Stockmann et al., 2013a). At DIV 42 stage of *in vitro* human MN^WTFUS^ cultivation, immunolabeled FUS localized strongly at the nuclei, and additional FUS punctae, overlapping with reference synaptic proteins Bassoon(N) and Homer1, were identified along the neurites (Figure 1A). The presence of FUS in the Synaptosomes of these neurons was confirmed by subcellular fractionation studies (Supplementary Figure 1A). However, these cells did not show a presence of the PSD marker protein PSD95 in the neurites (Figure 1B). Further, ultrastructural analysis of human MN^WTFUS^ showed that axons are clearly visible, synaptic contact is established and at the presynaptic terminal smaller clear vesicles (SCVs) and dense core vesicles (DCVs) are found, but also confirmed that at DIV 42 a mature postsynaptic density regions with a clear thickening of the postsynaptic membrane could not be detected, demonstrating immature synapses (Supplementary Figure 2).

The FUS antibody used in this study (RRID:AB\_1849181) has been tested previously (Neumann et al., 2009; Qiu et al., 2014; Sephton et al., 2014; Schoen, 2017). However, to further confirm the synaptic staining in other models, we performed immunohistochemical staining on mouse sections obtained from the lumbar part of the spinal cord. Besides its clear nuclear localization, FUS was present in neurites coinciding with Bassoon and VAChT punctae, ruling out any possible cell line specific bias of the FUS antibody (Supplementary Figure 3). Further trials with an alternative FUS antibody (RRID:AB\_309445) showed a similar subcellular distribution of FUS in motoneuron cells (Supplementary Figure 4).

### SR Microscopy Shows a Postsynaptic Localization of FUS in hiPSC Derived Control Motoneurons

**FIGURE 2:**
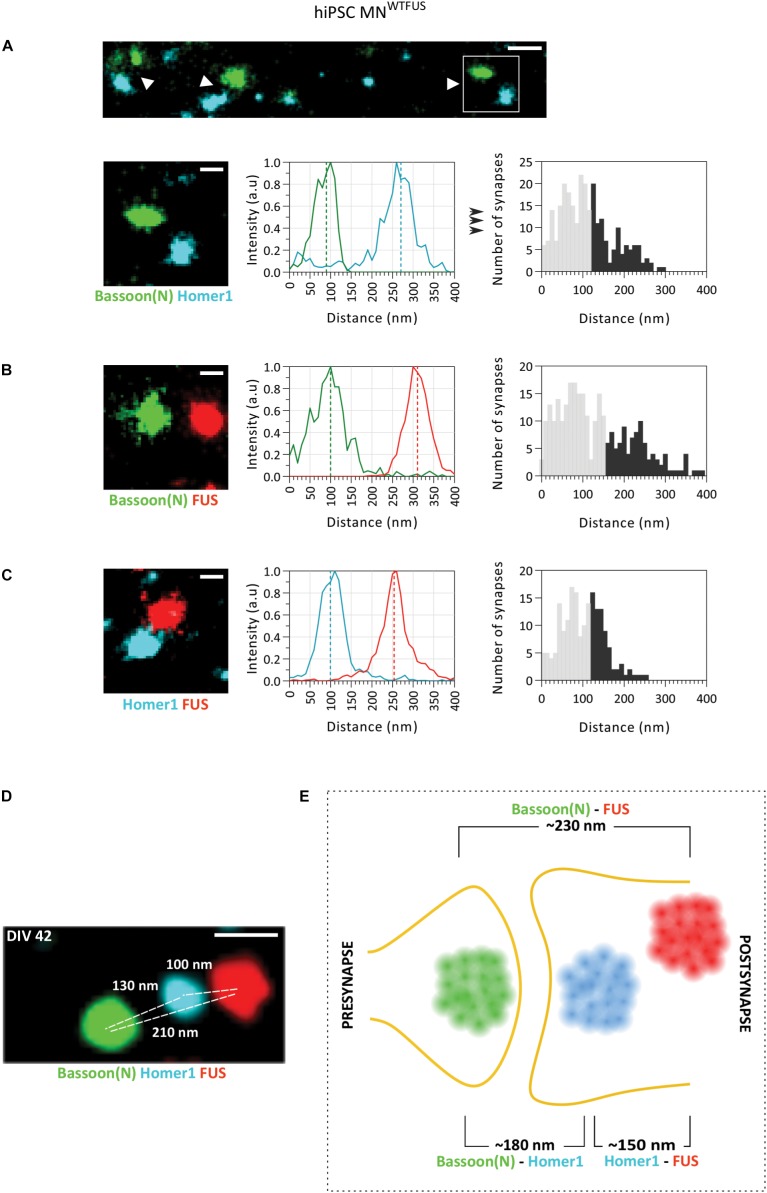
SMLM image analysis of synapses indicates a postsynaptic FUS in hiPSC derived MN^WTFUS^. **(A)** The top row shows an overview of synapses (white arrowheads) labeled for marker proteins Bassoon(N) and Homer1. Magnified view (white box marked in the overview) of a single synapse (second row, left-most panel) is shown, where the difference between the center of mass of the two populations intensities (indicated here by the dotted line in the middle panel) gives the distance between the protein clusters. The rightmost panel shows a histogram of such distances computed between Bassoon(N) and Homer1 from several synapses. **(B,C)** Representative synapse, intensity profile for each protein population, and computed histogram of the measured distances are shown also for **(B)** FUS and presynaptic Bassoon(N) and **(C)** FUS and postsynaptic Homer1. **(D)** Three-color SMLM image shows distribution of synaptic protein clusters within a single synapse. **(E)** Schematic representation of a human motoneuron synapse shows the mean approximate distances measured between different protein clusters at DIV42, as described in the text. All images are from cells fixed at DIV 42. For all histograms the lower 60% of the distances are shown in gray and the upper 40% in black. Scale bar is 200 and 100 nm for the overview and single synapses, respectively.

Next, to determine the specific synaptic compartment in which FUS localizes in human derived motoneurons we utilized the SMLM technique, since the diffraction limited resolution (about 200 nm) offered by conventional microscopy makes it difficult to distinguish between pre- and postsynaptic regions. First as a control, human MN^WTFUS^ cells were immunolabeled for the known synaptic markers Bassoon and Homer1 and were imaged using the dSTORM method. The reconstructed image in Figure 2A shows closely localizing Bassoon(N) and Homer1 populations, indicating synapses. Similarly, colocalizing populations were identified in neurons labeled for FUS with either of the synaptic markers (Figure 2B,C).

Next, to identify the mean position of FUS within the synapse, the quantitative distance information between FUS and the synaptic marker proteins was obtained from the SMLM images. The distance between two proteins was computed from the difference between the COM of their respective localization (as described in the section “Distance Analysis for Synaptic Proteins,” Figure 1A). For each pair of protein labeling, distances from more than hundred synapses were determined separately, and displayed in histograms with 10 nm bin steps. While the large number of single molecule localizations obtained for every image makes the distance determination of each synapse precise (better than 10 nm), the histograms showed a comparatively large distribution of the distances measured between the synaptic proteins. This broadening can be understood when considering that the three-dimensional synaptic structures have been projected into a two-dimensional image. Therefore, synapses whose trans-synaptic axis is directed out of the imaging plane (“face-view”) will always show overlapping protein localizations, while synapses which are orientated with their trans-synaptic axis in the focal plane (“side-view”) will display the largest possible distance (hence the accurate distance) between the synaptic proteins (Supplementary Figure 5). To account for this projection error, while also considering the biological variations within synapses, only the top 40% of the largest measured distances were used in the analysis. While this established procedure cannot accurately determine the precise distance for each protein pair, since the 40% threshold value was chosen rather arbitrarily, quantitative comparisons between different extracted distances are not affected by the precise choice of the threshold (method previously described in Schoen et al., 2016). The mean distance between synaptic marker proteins Homer1 and Bassoon(N) determined in this manner was ∼180 ± 4.3 nm (approximate distance ± standard error of mean from *n* = 272 synapses), which corroborates with previously published values from hippocampal neurons (Dani et al., 2010). The mean distance between FUS and the presynaptic marker Bassoon(N) was ∼230 ± 5.6 nm (*n* = 283 synapses) and that of FUS with postsynaptic marker Homer1 was ∼150 ± 3.6 nm (*n* = 189 synapses). Since FUS localizes nearer to Homer 1 than Bassoon, the comparison of these three distances establishes that FUS shows a postsynaptic localization in human MN^WTFUS^.

To further confirm the respective localization of Bassoon(N), Homer1, and FUS, another approach was sought, which would allow for the concurrent localization of these proteins within a single synapse. For this we used the recently developed Exchange-PAINT technique, which allows for multiplexing and enables SMLM imaging of three (or more) proteins within the same sample. Figure 2D shows an exemplary synapse imaged with this technique, where FUS is localized in the postsynaptic area behind the Homer1 cluster and away from the synaptic cleft, while the Bassoon(N) cluster marks the presynaptic area. Keeping in mind the above described expected variations in distances between individual synapses, the protein distribution is similar to the measured mean distances in the two-color SMLM experiments. To summarize the localizations, the computed mean distances are represented in a schematic (Figure 2E), showing a postsynaptic clustering of FUS in control human motoneurons.

### FUS Localizes in the Presynaptic Compartment of Mature Primary Rat Motoneuron Synapses

Since a high quality long-term cultures of hiPSC-derived motoneurones for SR imaging was challenging, we used rat primary cultures to analyze neurons in a more advanced stage of maturation. We performed analysis of synaptic FUS localization in rat primary motoneurons (rat MN^WTFUS^) at DIV14, as similar culture times have previously been used in other studies (Martinou et al., 1989; Stockmann et al., 2013b; Schoen et al., 2016). In our cultures, upon labeling these cells at DIV 14 with FUS and multiple other synaptic marker, we could visualize colocalizing populations along the neurites (Supplementary Figure 7). Presence of FUS in the synaptosomal region of rat MN^WTFUS^ was further confirmed by subfractionation experiments (Supplementary Figure 1C).

**FIGURE 3:**
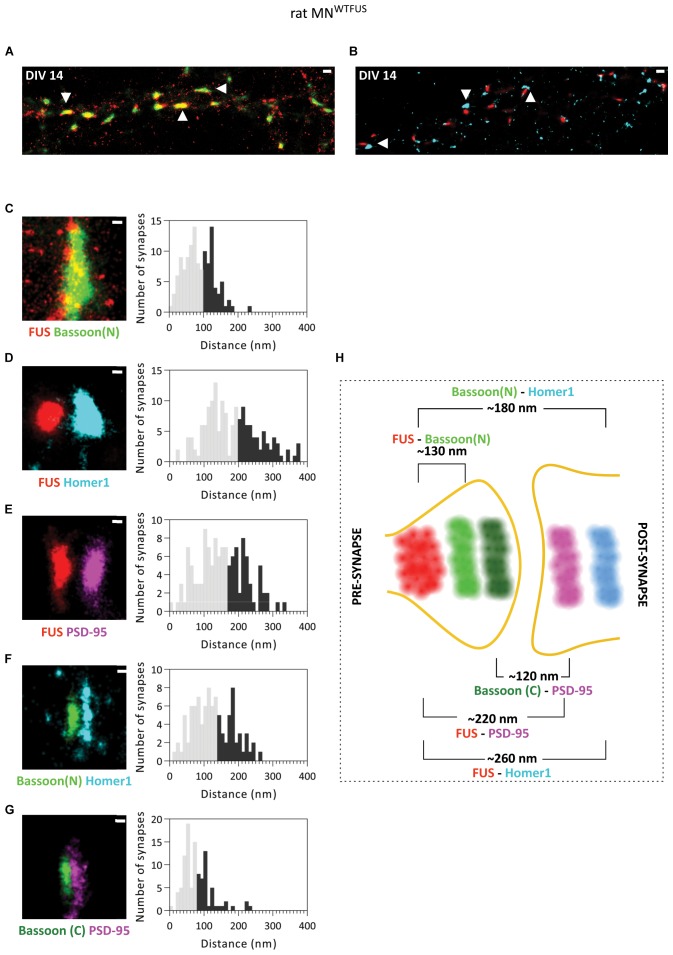
Primary rat MN^WTFUS^ at DIV 14 shows FUS clustering in the axonal compartment of mature synapses. An overview of mature synapses (examples marked with white arrowheads) observed in rat primary motoneurons at DIV 14 with SMLM imaging, labeled for FUS and **(A)** presynaptic protein Bassoon(N), or **(B)** postsynaptic marker Homer1. **(C–G)** Images of single synapses at DIV 14 (left) for labeling of **(C)** FUS and Bassoon**, (D)** FUS and Homer1, **(E)** FUS and PSD95, **(F)** Bassoon(N) and Homer1, and **(G)** Bassoon(C) and PSD95. The right panels show histograms of distances between the COM of the respective protein clusters. The lower 60% of the distances are shown in gray and the upper 40% in black. **(H)** An overview schematic of a motoneuron synapse shows the mean distances between different synaptic proteins, and a presynaptic FUS clustering in mature synapses of rat motoneurons. Scale bar is 500 nm for the overview and 100 nm for the single synapses.

At DIV 14 rat MN^WTFUS^ cells were then imaged with dSTORM technique to determine the specific synaptic compartment within which FUS localizes. Overview images show synapses with a close proximity between FUS and presynaptic Bassoon(N), while in most synapses the FUS and Homer1 clusters were seperated by a gap (Figure 3A,B). Further statistical analysis revealed a mean distance of 130 ± 3.5 nm (*n* = 124 snyapses) between FUS–Bassoon(N), while a bigger distance of 260 ± 6.1 nm (*n* = 158 synapses) was computed for FUS–Homer1 labeling, revealing a presynaptic clustering of FUS in this case (Figure 3C,D). To confirm the presence of a PSD and to get an overview of a mature motoneuron synapse, we also mapped the distances for other synaptic protein in the rat MN^WTFUS^ (Figure 3E–G). The reconstructed images showed large colocalizing populations of synaptic proteins, where a bar-like structure was observed for the scaffolding proteins Bassoon, Homer1, and PSD95. The relative distance between the localizations of FUS and PSD95 was found to be 220 ± 6.3 nm (*n* = 126 synapses). While analysis of synaptic markers Bassoon(N) and Homer1 revealed a mean distance of 180 ± 5.4 nm (*n* = 97 synapses). Further, a distance distribution with a mean of 120 ± 6 nm (*n* = 104 synapses) between their respective COM was calculated for PSD95 and C-terminally labeled Bassoon(C). A schematic summarizes the results of our SMLM-based mean distance calculations and gives a good impression of the synaptic architecture of a typical motoneuron synapse (Figure 3H). The difference in the relative position of the N- and C-terminal antibody observed in the schematics can be attributed to an elongated positioning of the Bassoon protein across the presynaptic compartment, which has been previously reported in hippocampal synapses (Dani et al., 2010).

Additional analysis of rat MN^WTFUS^ cells, at a more mature stage of DIV 21, showed an identical distribution of synaptic proteins and confirmed a presynaptic localization of FUS in mature synapses (Supplementary Figure 6).

### FUS Shows a Variation in Localization in Developing/Immature Primary Rat Motoneuron Synapses

**FIGURE 4:**
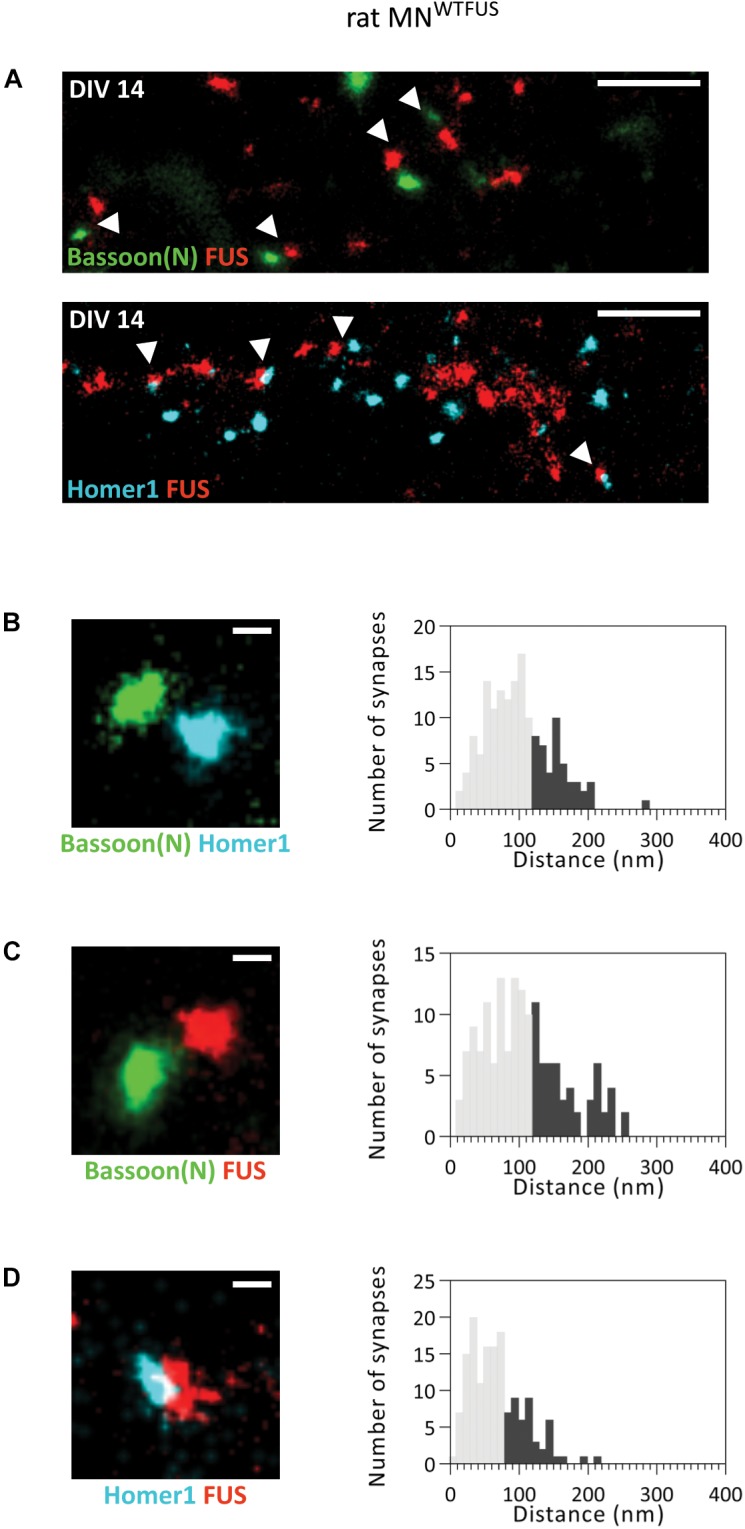
FUS shows a postsynaptic accumulation in immature (smaller) synapses of rat MN^WTFUS^ at DIV 14. **(A)** The overview SMLM images shows several small synapses (marked with white arrowheads) labeled for Bassoon(N), FUS, and Homer1. **(B–D)** Exemplary images of individual synapses and histograms for the analyzed distances between the COM of the two protein populations are shown for **(B)** Bassoon(N) and Homer1, **(C)** Bassoon(N) and FUS, and **(D)** Homer1 and FUS. The lower 60% of the distances are shown in gray and the upper 40% in black. Scale bar is 500 nm for the overview and 100 nm for single synapses.

Our analysis of FUS localization to specific regions within the synapse in rat MN^WTFUS^ places FUS in the presynapse, similar to that noted previously in rat hippocampal neurons (Schoen et al., 2016), and in contrast to our findings of a postsynaptic localization in human MN^WTFUS^. Here the conclusion could be that either a pre-snyaptic FUS localization is species dependent, being observed only in rat neurons, or that further maturation-based analysis is needed. To this end, upon comparing the images of synapses from the rat and human models, we found a considerable differences in the sizes of their synaptic protein clusters. While in the human MN^WTFUS^ synapses the Homer1 protein clusters have an average area of 0.008 ± 0.0006 μm^2^, the measured synaptic clusters in rat motoneurons are much larger with an average area of 0.06 ± 0.004 μm^2^ (Supplementary Figure 7). Interestingly, in the rat MN^WTFUS^ at DIV14 we observed in addition to the above discussed large synapses, several colocalizing synaptic protein clusters, with an area <0.03 ± 0.001 μm^2^, indicating the presence of smaller sized synapses (Figure 4A). An overview of the images indicated that bigger synapses were always found in the neurites closer to the motoneuron soma, while small synapses were found more toward the neurite perifery, with an increasing disparity appearing with longer cultivation time (Supplementary Figures 7A,B). The smaller synapses were found to have dimensions comparable to that of the human MN^WTFUS^ (Supplementary Figure 7C). Hence in the rat motoneuron MN^WTFUS^ culture at the same culture-stage there were present both small (<0.03 μm^2^) and large synapses, which were easily distinguished with the sub-diffraction resolution offered by the SMLM.

We then analyzed the localization of FUS within these small synapses and measured a distance of ∼160 ± 4.65 nm (*n* = 157 synapses) between the pre- and postsynaptic marker Bassoon(N) and Homer1 (Figure 4B). This distance matches to that observed for the localization of these proteins in human MN^WTFUS^. Hence, there is good evidence that these colocalizing populations correspond to an intact and active synaptic structure. Further analysis showed a mean distance of ∼180 ± 5.4 nm (*n* = 153 synapses) between FUS and Bassoon(N) (Figure 4C). While the FUS and Homer1 populations appear to overlap and show a shorter distance of ∼130 ± 2.8 nm (*n* = 150 synapses) (Figure 4D). Thus, in contrast to the presynaptic localization of FUS in large synapses of rat motoneurons, there exists at the same time a class of smaller synapses, possibly either developing/immature, where FUS localizes to the postsynaptic side.

To further elaborate on the change in FUS localization during maturation we imaged rat MN^WTFUS^ at different developmental stages from DIV1 to DIV21 (Supplementary Figure 8). Both FUS and Bassoon could be immunolabeled in the motoneurons already at DIV2 and small clusters could be visualized along both dendrites and axons, while Homer1 labeling was possible from DIV5. But distinctive synapses, with colocalizing presynaptic Bassoon and postsynaptic Homer1 populations, were observed only from DIV14 (Supplementary Figure 8A). From DIV2 onward it was possible to distinguish between motoneuronal axons labeled with NF-H antibody, and dendrites specifically marked by the MAP2 antibody. Data from these stages showed that FUS-positive punctae mostly localized in the dendrite, while a relatively minor fraction of FUS punctae were seen along the axons (Supplementary Figure 8B).

### FUS and Exemplary Synaptic Proteins Show Accumulation in ALS Patient Derived Motoneuron Synapses

**FIGURE 5:**
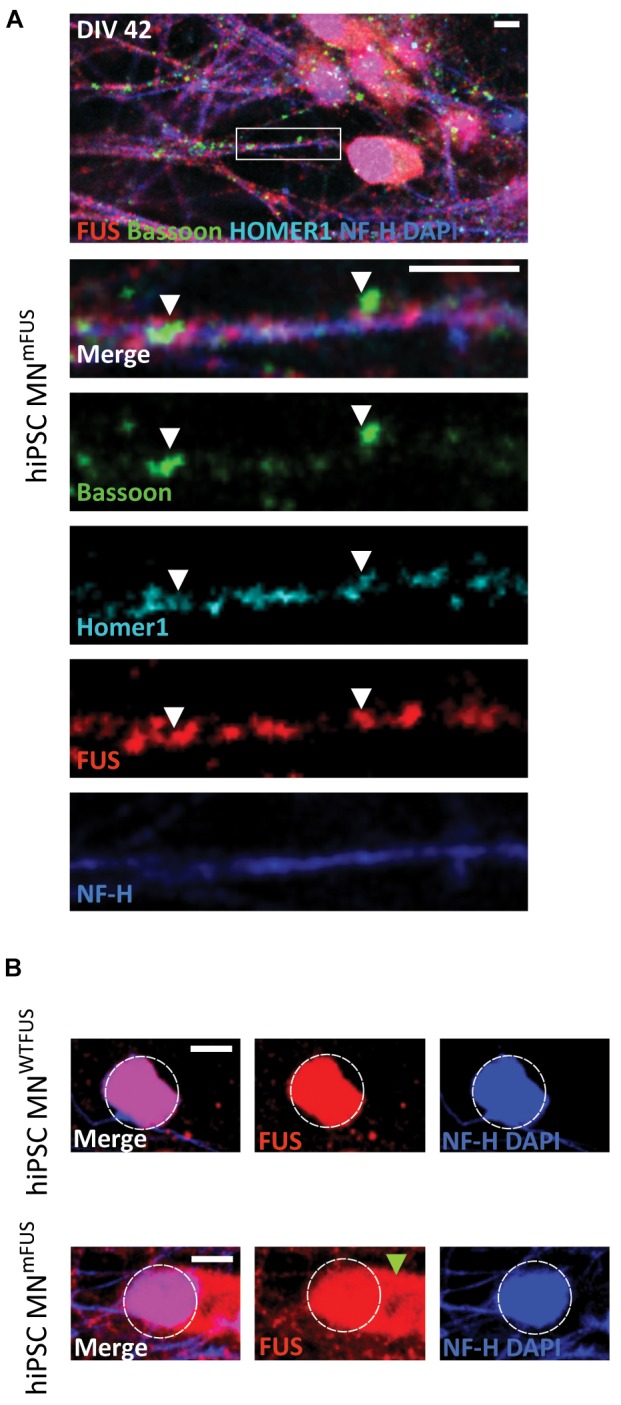
ALS patient derived hiPSC motoneurons show mislocalization of mFUS in the cytoplasm. **(A)** Human MN^mFUS^ cells identified by the NF-H (blue) labeling of the axon were imaged with a confocal microscope. The lower rows depict the magnified crop (marked by white box in the overview) as merged or single channel images. Immunolabeled FUS (red) shows a subcellular location along the neurites overlapping with the other presynaptic marker Bassoon (green) and postsynaptic marker Homer1 (cyan) indicated by the white arrowheads. **(B)** Comparison between human MN^WTFUS^ cells (upper) and human MN^mFUS^ cells (lower) labeled for FUS, NF-H, and DAPI. The white dotted circle approximately outlines the nucleus and the green arrowhead points to the cytoplasmic clusters of mFUS in patient derived cells. All scale bars 5 μm.

Since FUS mutations have been linked to ALS, we next wanted to compare the proteins distribution as obtained by dSTORM imaging in control and patient motoneurons. We used an ALS patient derived cell line, which carries a severe frameshift FUS mutation in exons 14 and 15 within the protein-coding region. These Asp502Thrfs^∗^27 cell line and differentiated motoneurons culture have been previously described in detail (Higelin et al., 2016). The motoneurons (human MN^mFUS^) derived from this patient cell line were also cultured till DIV42, when FUS punctae overlapping with synaptic markers was observed along the neurites (Figure 5A). Presence of FUS in Synaptosomes was confirmed by subfractionation studies (Supplementary Figure 1A). Moreover, as has been previously reported, mislocalization of mFUS to the cytoplasm was also observed in the patient motoneurons (Figure 5B). Further ultrastructural analysis of patient derived motoneurons showed presynaptic compartments containing SCV, DCV, and not fully developed PSDs similar to control cells, but additional abnormal structures, probably stress granules (Higelin et al., 2016), were also detected at axonal and cytoplasmatic level (Supplementary Figure 2).

**FIGURE 6:**
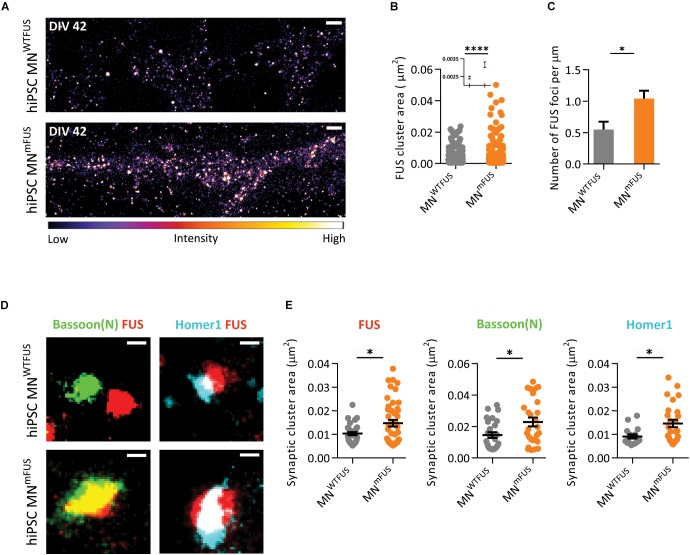
ALS patient derived human MN^mFUS^ shows increased expression and accumulation of mFUS. Data obtained from SMLM analysis of motoneuronal cells derived from ALS patients with FUS mutation were compared to control cells derived from healthy human volunteers. **(A)** Exemplary images of control (top) and ALS patient cells (bottom) immunolabeled with anti-FUS antibody. The calibration bar shows the color scheme based on intensity. **(B)** Scatter plot comparing the cluster sizes of FUS in control (gray) and patient cells (orange). Inset shows the mean and SEM of each population. **(C)** Comparison of the number of FUS clusters along neurites per μm in patient and control cells. **(D)** Exemplary synapses from the human MN^WTFUS^ and human MN^mFUS^ cells co-stained for FUS, Bassoon(N), and Homer1. **(E)** Scatter plots comparing the area of synaptic protein clusters of FUS, Bassoon(N), and Homer1 show an increased accumulation of synaptic proteins in patient motoneuron synapses compared to control. The black line marks the mean and error bars represent SEM. Unpaired *t*-test conducted to compare control vs. patient data shown by ^∗^ above the graphs. ^∗^*p* ≤ 0.05 and ^∗∗∗∗^*p* ≤ 0.0001. Scale bar 1000 nm for the overview and 100 nm for all single synapses.

With SMLM in the human MN^mFUS^ cells we observe a general increase in mFUS clusters in neurites compared to FUS clusters in control cells (Figure 6A). For further analysis, we determined the area of these FUS-positive clusters from the SMLM images (the section “Cluster Area Analysis”). The resulting scatter plot of the cluster area shows a large distribution, with 95% of FUS clusters showing a size <0.01 μm^2^ in both control and patient cells (Figure 6B). These small FUS-positive punctae were present everywhere along the neurites in both cell types, but showed an increased density in the patient cells. While the human MN^WTFUS^ had an average of 0.54 ± 0.013 of these FUS-positive punctae per μm, human MN^mFUS^ cells had 1.04 ± 0.120 per μm (Figure 6C). The remaining 5% population of FUS clusters had a larger area (>0.01 μm^2^), and probably corresponded to synaptic FUS. To further elaborate on that, we labeled the cells with FUS and markers Bassoon(N) or Homer1 antibodies to study the synaptic clustering of these proteins (Figure 6D). The cluster area of individual synapses, showing colocalizing populations of FUS and either of the synaptic marker Bassoon(N) or Homer1, was analyzed (as described in the section “Cluster Area Analysis”). While there was a rather wide distribution of the synaptic cluster areas a clear trend between patient and control cells was observed, where all three proteins showed an increased accumulation in patient synapses (Figure 6E). The average cluster areas are increased by 43% for synaptic FUS, while there was an increase of 56% and 60% in the synaptic clustering of Bassoon(N) and Homer1, respectively, in patient cells. Due to this aberrant accumulation of mFUS at synaptic sites, it was impossible for us to determine a precise pre- or postsynaptic localizations in the patient cells.

## Discussion

### FUS Localization in Synaptic Compartment Is Maturation Dependent

In this study we have used SMLM to study the synaptic localization of ALS-associated protein FUS in motoneurons. A typical synapse is a small structure, where the distances between the presynaptic terminal and the postsynapse are much smaller than the diffraction limit of light, making it difficult to resolve them using conventional fluorescence microscopes. Therefore, though confocal imaging and biochemical subfractionation results suggested presence of FUS in synapses, neither of the approaches were precise enough to discern the synaptic compartment in which FUS localizes. However, recently a growing number of studies are using SR microscopy to visualize the complex sub-diffraction level structures in synapses, and the single molecule localization technique is especially suited for neuronal imaging as it allows visualization of immunolabeled proteins with the highest spatial resolution (Sydor et al., 2015; Heller and Rusakov, 2017). Hence, the technique was chosen for this study, and to the best of our knowledge, we are the first to resolve the protein architecture of a motoneuron synapse with sub-diffraction level fluorescence microscopy.

We showed that FUS is present in the synapses of human MN^WT^, and that in these cells it localizes within the dendritic compartment close to the postsynaptic marker Homer1. On the contrary, imaging in rat MN^WTFUS^ revealed a more presynaptic localization of FUS. To explain this discrepancy in the localization, we compared the synapses of these two models and found several differences. The protein cluster area in most rat MN^WTFUS^ synapse was significantly larger compared to the human MN^WTFUS^. Additionally, these bigger synapses in rat motoneurons showed a bar-like distribution of the scaffolding proteins Bassoon, PSD-95, and Homer1, similarly to that observed previously by other studies in mature hippocampal synapses (Dani et al., 2010; Tao-Cheng et al., 2014). On the other hand, in human synapses the proteins Bassoon and Homer1 did not display such a bar-like structure, but rather had a smaller and rounded distribution. Similarly, we were unable to label human synapses at DIV42 with a PSD95 antibody, and ultrastructure data acquired from EM also showed no thickening of the postsynaptic neuron to form a PSD. This indicates an absence of a mature postsynapse formation, and points to a “developing or immature” stage of the neurons. Our further analysis also revealed a sub-population of smaller synapses in the rat MN^WTFUS^, with dimensions comparable to the human MN^WTFUS^ synapses, and a similar postsynaptic FUS accumulation. The small synapses from both rat and human derived cultures showed comparable smaller distribution area of the PSD-associated protein Homer1, while the big rat synapses showed a larger cluster area. These results are consistent with EM studies of hippocampal synapses, which show that area and thickness of the PSDs are significantly larger in mature synapses compared to the developing ones (Grabrucker et al., 2009). Hence, we infer that both the smaller synapses in rat motoneurons and the synapses in hiPSCs derived motoneurons are in an immature/developing stage, while the larger structures in rat motoneurons correspond to mature synapses. Furthermore, we could only identify the first synapses with colocalizing pre- and postsynaptic proteins after day 14 in rat motoneuronal cultures, making it clear that the cells at this time-point are still undergoing synaptogenesis and have synapses in varying developmental stages (Beaudet et al., 2015). The presence of postsynaptic FUS, in the immature synapses of both human and rat neurons, points to a maturation dependent localization that is not species specific. We can therefore summarize that FUS shows a varying synaptic location based on maturity, with clustering in dendritic spine observed during development and localizing to the axonal compartment of mature synapses.

The dynamic maturation-based localization, we observed, could help to explain contradictions in the FUS localization results obtained from previous brain sub-cellular fractionation studies that showed association of FUS with the PSD (Aoki et al., 2012), and the SMLM imaging data that localized FUS to the presynaptic region (Schoen et al., 2016). Recent research describes the presence of “immature” synapses in mouse brain sections, where they could be distinguished by a comparatively smaller “Homer population” (Dani et al., 2010). As Aoki et al. (2012) used a whole brain lysate in their synaptosomal fraction, which possibly also include immature synapses, the postsynaptic FUS association they observed can be explained.

Although the specific function of FUS in the synapse is not known, several studies have shown its involvement in both dendritic- and axonal-related activities. In the dendrites, FUS is important in the spine formation, as rodent models with FUS mutations have shown a reduction in dendritic arbors, a stunted postsynapse development, and synaptic defects (Fujii et al., 2005; Sephton et al., 2014; Qiu et al., 2014; Ishigaki et al., 2017). Further, FUS depletion is also known to cause reduced expression of GluA1, an AMPA receptor subunit in the postsynapse, which can affect the synaptic strength (Udagawa et al., 2015; Yokoi et al., 2017). This involvement of FUS in dendritic development would explain its presence in the postsynapse during the early stages of maturation, which we observed in this study. Similarly in relation to its presynaptic role several groups enumerate the importance of FUS in axonal functions. In rat hippocampal neurons, FUS localizes to the synaptic vesicles (Schoen et al., 2016), where its involvement in the local translation machinery and the possibility of a putative exacerbated presynaptic local translation as a result of increased presynaptic FUS accumulation in diseased states have been proposed (Schoen, 2017). Further, mFUS is reported to cause axonal transport defects in motoneurons (Guo et al., 2017), and presynaptic abnormalities in mice models (Sharma et al., 2016). Drosophila models expressing mutant FUS also experience a subsequent decline in axonal transport (Baldwin et al., 2016). Additionally, this decline was observed to be more rapid in adult drosophila mutants compared to the larva, pointing to a possible maturation-based function of FUS in the axonal compartment. Recently, Shigeoka et al. (2016) performed an elegant axonal specific translatome study in mouse, showing a variation in axonal mRNAs and in local protein synthesis control, based on the development stage. They further propose that such a dynamic translation control might help to remodel the proteome locally based on extrinsic cues (Shigeoka et al., 2016). Hence, FUS with its possible role in regulating local translation might be expected to vary in axons based on the neuronal maturation stage, consistent with our observations. Other related proteins such as TDP-43 and Fragile X Mental Retardation Protein (FMRP) have also been localized in axons as well as dendrites (Ling, 2018), linking the involvement of RNA-binding proteins in both pre- and postsynaptic mRNA regulation.

While the exact mechanism of how FUS changes the clustering position from the dendritic spine to the axonal bouton during maturation is beyond the scope of this study, we can speculate about some probable mechanisms. It is possible that FUS could be transported to both synaptic compartments, but during development clusters preferentially in the postsynapse, while upon maturation it shows accumulation only in the axonal compartment. The presence of FUS transport granules in both axon and dendrites, which we observed in developing rat motoneurons, lends support to this hypothesis. In another possibility, the observed difference in FUS localization could also be caused by a trans-synaptic propagation of FUS during maturation. Such a transport was observed, for example in drosophila, where the Arc1 protein is known to form a capsid-like structure that binds to mRNA, and is then transported by extracellular vesicles across the synaptic gap (Ashley et al., 2018). Interestingly, recent research discuss about trans-neuronal spread of FUS and the other ALS-associated protein TDP-43 (Braak et al., 2013; Ravits et al., 2013; Schoen et al., 2016). In the case of TDP-43, which has structural and functional similarities to FUS, a bi-directional transfer was observed from the axonal terminal of cortical neurons (Feiler et al., 2015). Whether any such mechanisms of FUS transfer across the synaptic membrane, in a maturation-dependent fashion, are involved in motoneurons, and what contribution they might have on the suspected prion-like propagation of ALS disease, would be an interesting future study.

### Mutant FUS Shows Increased Clustering in the Neurites and Synapses of ALS Patient Motoneurons

In the second part of this study we imaged mutant FUS localization in ALS patient derived motoneurons where, as has been previously reported, we observed mislocalized mFUS clusters in the cytoplasm of patient cells. In the acquired SR images, we additionally observed an almost twofold increase of FUS-positive clusters in the neurites of ALS patient cells compared to control. As mutant FUS is known to induce axonal and dendritic transport defects in neurons, it is quite conceivable that an increased clustering could be one of the contributing factors (Shahidullah et al., 2013; Guo et al., 2017).

We also measured an increased accumulation of FUS in the synapses of the patient cells when compared to control motoneurons. As mFUS displays a loss of regulatory control of mRNA stability, and such neurons are known to have synaptic dysfunctions (Ishigaki and Sobue, 2018; Ling, 2018), the increased mFUS accumulation at the synapse could contribute to these alterations by interfering with local mRNA control. We observed that both Bassoon(N) and Homer1 also show increased clustering in the synapses of patient motoneurons. It is suggested that FUS aggregates might act as a “seed” and sequester other essential proteins in the neuron to cause further protein aggregation (Sephton and Yu, 2015; Ratti and Buratti, 2016; Khalil et al., 2018). Taken together our data suggest that the higher accumulation of other synaptic proteins may be a result of this “sequestering” property of FUS. Although both loss-of-function and gain-of-function hypotheses for FUS dysfunction in ALS have been proposed, a loss of function in RNA regulation and a toxic gain of function at synapses are suggested (Sephton and Yu, 2015; Ishigaki and Sobue, 2018; López-Erauskin et al., 2018). The increased accumulation of proteins in synapses of mutant FUS cells might fit in with a synaptic gain-of-toxicity hypothesis. However, as this study is from an *in vitro* model system, further experiments are required to confirm if such synaptic protein sequestering effect is also observed in *in vivo* conditions.

Motoneurons are complex cells with neurites that extend a long distance away from the soma. This creates a need for local synaptic translation that would allow for comparatively rapid synapse modifications in response to neuronal inputs (Taylor et al., 2016; Yasuda and Mili, 2016). The presence of mRNA, translation factors, and ribosomes needed for local protein synthesis in the dendrites has been well established, and recent studies have discovered a similar protein synthesis machinery in the axonal compartment (Cioni et al., 2018; Khalil et al., 2018). Given its synaptic localization in motoneurons, FUS is suspected to play a role in this local translation and hence, any mutation in FUS might contribute to synaptic dysfunctions. In neurodegenerative diseases like ALS synaptic defects occur before motoneuron degeneration (Sephton et al., 2014; Ratti and Buratti, 2016), making synaptic FUS and its increased accumulation an important target for possible therapies.

## Conclusion

Single molecule localization microscopy offers important insights into the synapse architecture by visualizing the protein distribution in the sub-diffraction level structures. The new Exchange-PAINT technique opens up the possibilities of labeling multiple proteins at the same time to observe various synaptic interactions. Here, using the SMLM techniques, we have shown that in motoneurons FUS is localized within different synaptic compartments based on maturity. We also found that mFUS in ALS patient derived neurons shows an increased accumulation at the synapse, along with the active zone protein Bassoon and the PSD-associated protein Homer1. Therefore, in future it will be interesting to study how this protein accumulation affects the synaptic functions in patient cells and contributes to the ALS disease progression.

## Ethics Statement

All procedures with human material: This study was carried out in accordance with the recommendations of the ethical committee of Ulm University (Nr.0148/2009 or 265/12) with written informed consent from all subjects. All subjects gave written informed consent in accordance with the Declaration of Helsinki. The protocol was approved by the “Declaration of Helsinki concerning Ethical Principles for Medical Research Involving Human Subjects.” All experiments involving animals: This study was carried out in accordance with the recommendations of the Federal Government of Germany, the National Institute of Health, and the Max Planck Society. The protocol was approved by the review board of the Land Baden-Württemberg (Permit Number O.103).

## Author Contributions

DD, MD, MS, TB, and JM designed and outlined the study. DD, JH, and MD did the rat preparation, cell culture, and confocal imaging. DD conducted the SMLM measurements and performed all the data analysis. TV provided support for the experiments. DD, MD, TB, and JM jointly wrote the manuscript.

## Conflict of Interest Statement

The authors declare that the research was conducted in the absence of any commercial or financial relationships that could be construed as a potential conflict of interest.

## Abbreviations

ALS: amyotrophic lateral sclerosis
COM: center of mass
DAPI: 4′,6-diamidino-2-phenylindole
dSTORM: direct stochastic optical reconstruction microscopy
EM: electron microscopy
FUS: fused in Sarcoma
hiPSCs: human induced pluripotent stem cells
human MN^mFUS^: human induced pluripotent stem cells derived motoneurons from ALS patient with an Asp502Thrfs^∗^27 FUS mutation, human MN^WTFUS^, human induced pluripotent stem cells derived motoneurons from control volunteers
MAP2: microtubule-associated protein 2
NF-H: neurofilament heavy
PSD: postsynaptic density, rat MN^WTFUS^, motoneurons derived from wild-type rat embryos
SMLM: single molecule localization microscopy
SR: super-resolution
Tdp-43: Tar DNA-binding protein 43
VAChT: vesicular acetylcholine transporter
