# Supplementary material for: Synaptic FUS Localization During Motoneuron Development and Its Accumulation in Human ALS Synapses
Source: Front Cell Neurosci. 2019 Jun 12;13:256. doi: 10.3389/fncel.2019.00256 (PMC6582137; doi:10.3389/fncel.2019.00256)
Supplement: Supplementary file 1 [file Data_Sheet_1.PDF]

Supplementary Material

Supplementary Figures

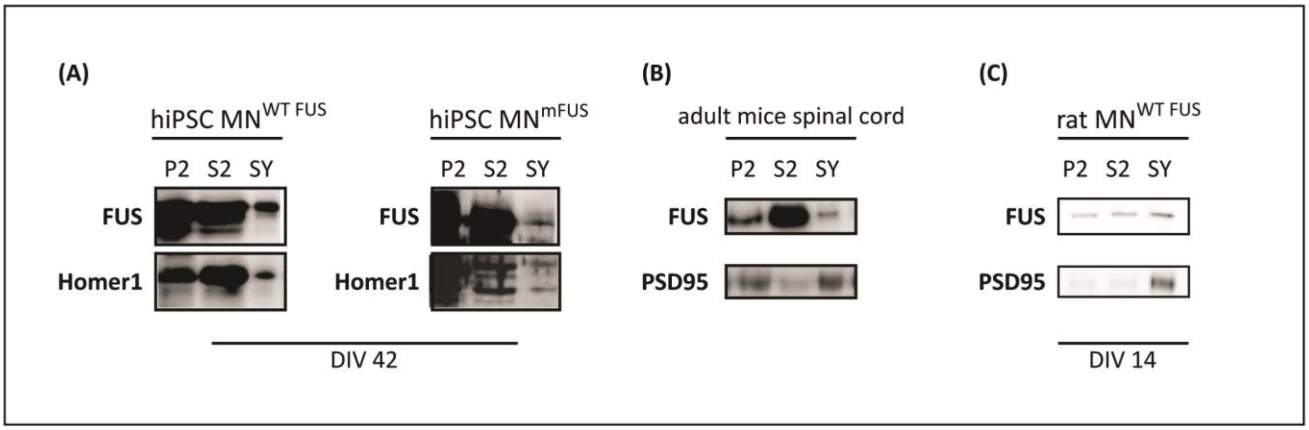

Supplementary Figure 1: FUS is present in motoneuron synaptosomes of different model systems. After biochemical fractionation, FUS and synaptic proteins (Homer1 and PSD-95) bands can be clearly identified in all fractions including synaptosomes. Data shown for **(A)** hiPSC derived motoneurons from both control and ALS patients **(B)** adult mice spine tissue and **(C)** embryonic rat derived primary motoneurons at DIV 14. P2 – crude membrane, S2-cytosolic compartment, SY-synaptosomal fraction.

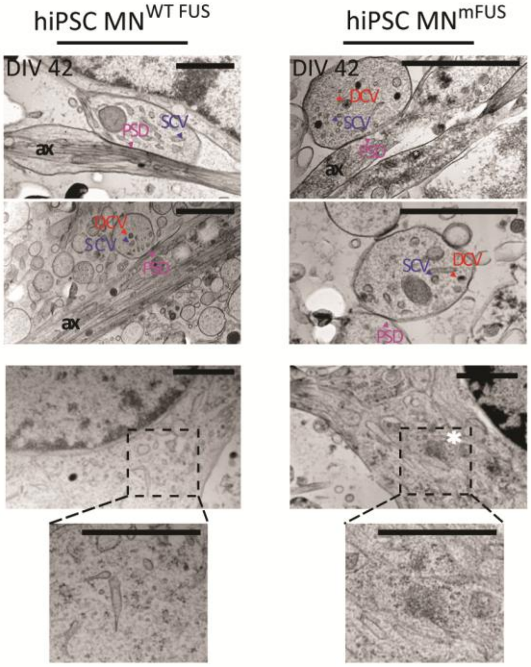

Supplementary Figure 2: Ultrastructure analysis confirms an immature post-synaptic structure in the human iPSC derived motoneurons. In the electron microscopy images axons (ax) of both, control (left column) and patient motoneurons (right column) at DIV 42, are clearly visible (uppermost panel) and synaptic contact is observed. At the presynaptic terminal smaller clear vesicles (SCV -blue arrows) and dense core vesicles (DCV - red arrows) are marked. A mature post synaptic density regions with a clear thickening of the post-synaptic membrane was not detected (magenta arrow). The magnified crop (dotted box) shows abnormal structures at axonal and cytoplasmatic level in patient cells (asterisk). All scale bars 500 nm.

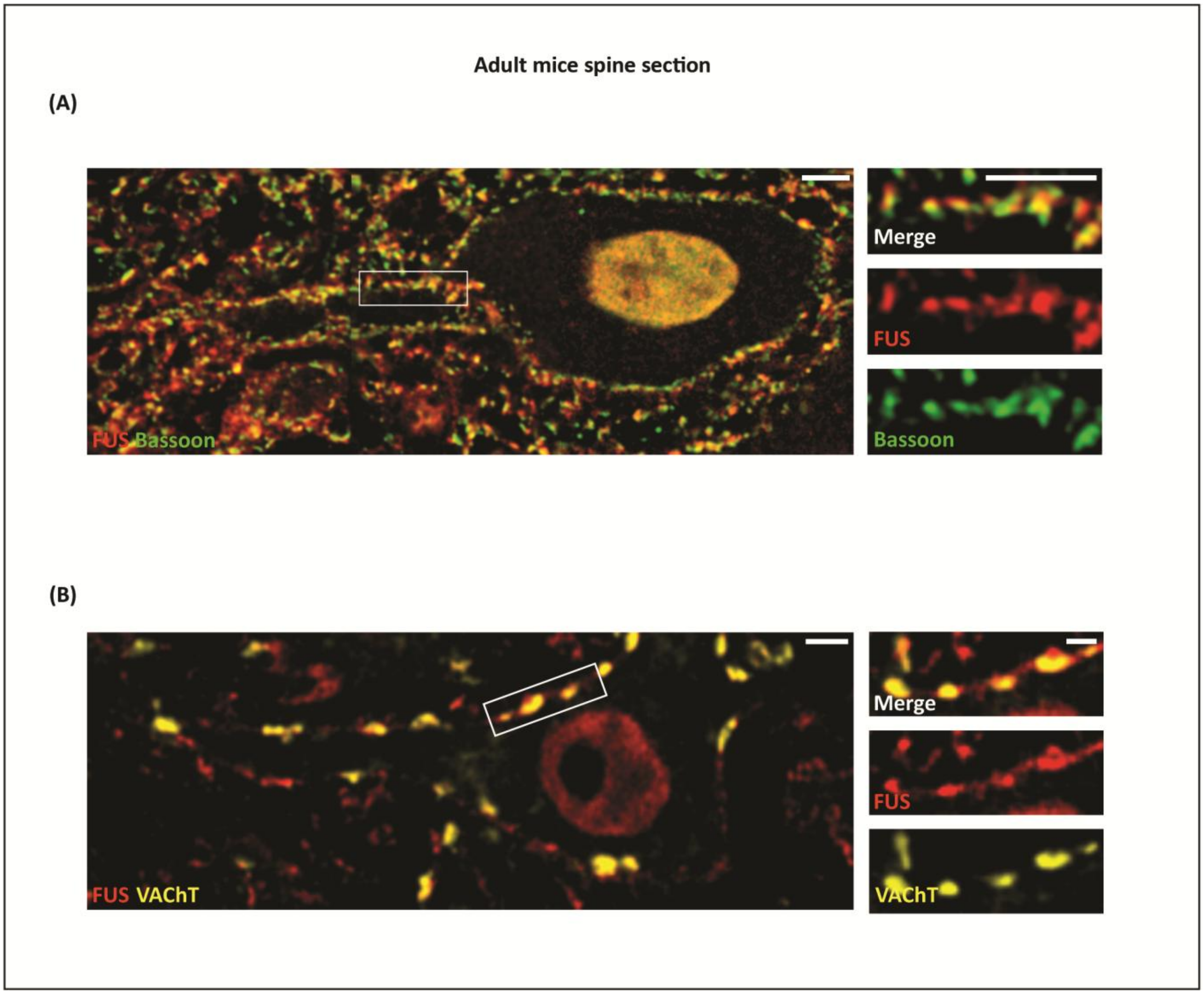

Supplementary Figure 3: Synaptic FUS observed in murine spine sections. Confocal images of motoneurons from the lumbar part of adult mice spinal cord (ventral horn). Immunofluorescence staining, for **(A)** FUS (red) and Bassoon (green) and **(B)** FUS (red) and VAChT (yellow), showed colocalizing population depicting synapses along the soma and neurites. VAChT - Vesicular Acethylcholine Transporter. All scale bars 5 μm.

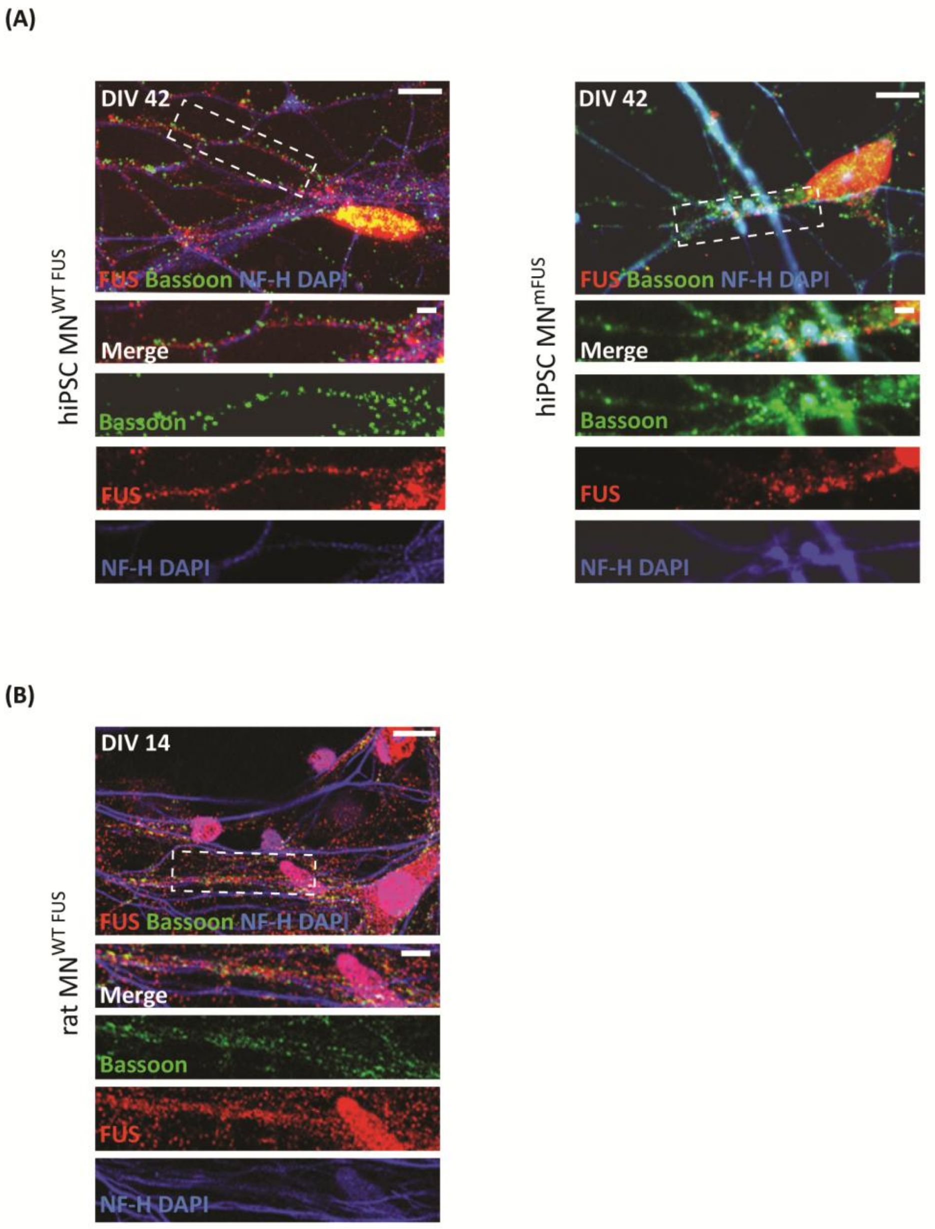

Supplementary Figure 4: Immunocytochemistry with an alternative antibody shows a comparable FUS distribution in motoneurons. (A) hiPSC derived motoneurons from both control (left) and patient (right) when labelled with FUS-antibody (Bethyl, RRID:AB_309445) show synaptic FUS colocalizing with Bassoon. **(B)** Similar synaptic FUS is observed in rat derived primary motoneurons. All scale bars 500 µm.

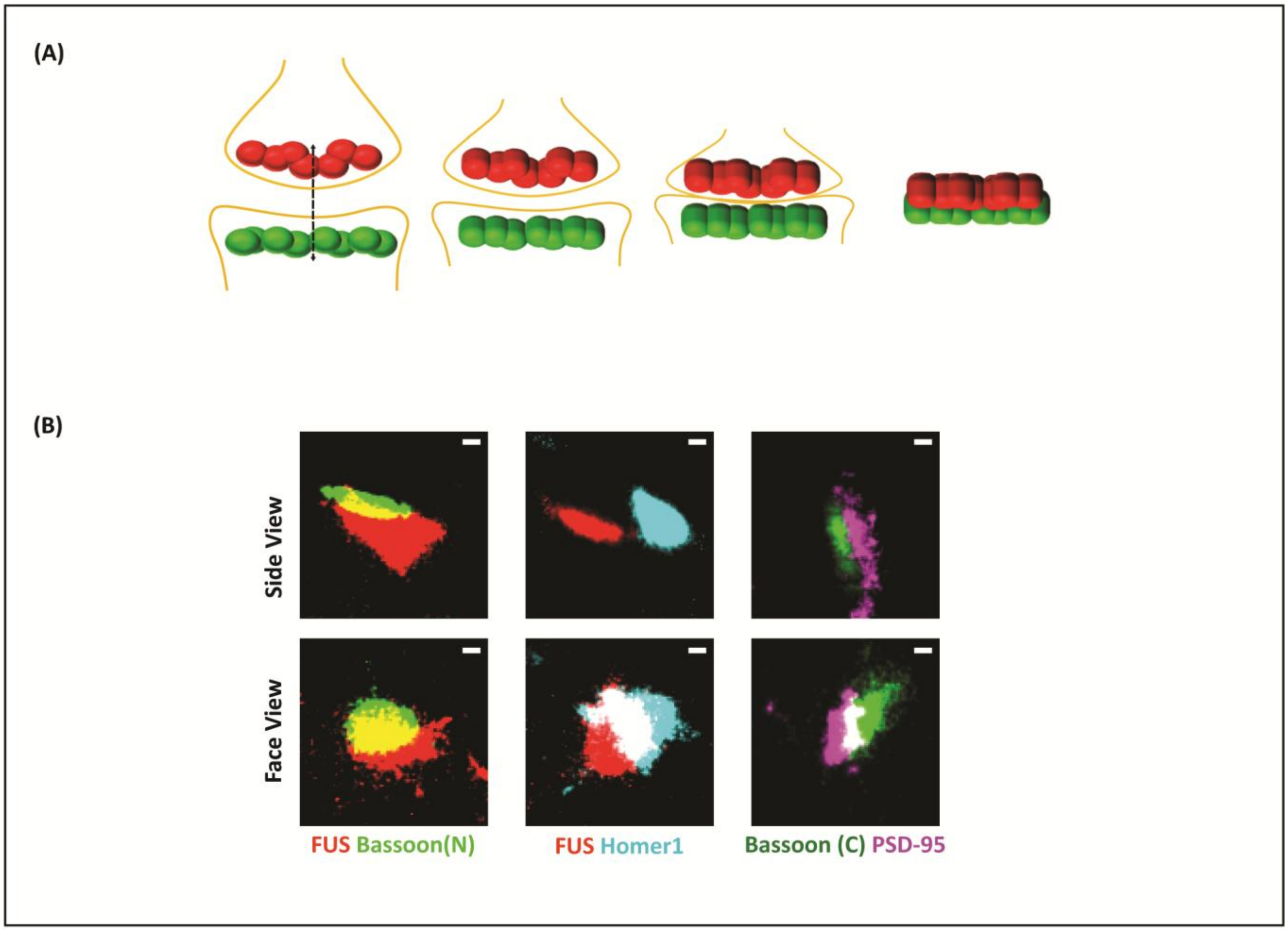

Supplementary Figure 5: Comparison of different synaptic orientations. (A) A schematic depicting the orientation of the synapses with respect to the focal plane. The protein populations show a varying degree of overlap, leading to a variation in the measured distances between them. The first sketch from the left shows a synapse with the trans-synaptic axis (dotted line) aligned in the imaging plane (side view) where the accurate distance between the two protein populations can be determined. **(B)** Representative synapses from rat motoneurons showing synapses aligned in a side-view (top row) and with face-view (bottom row) labelled for Homer1, Bassoon(N), Bassoon(C), FUS, and PSD-95. All scale bars are 100 nm.

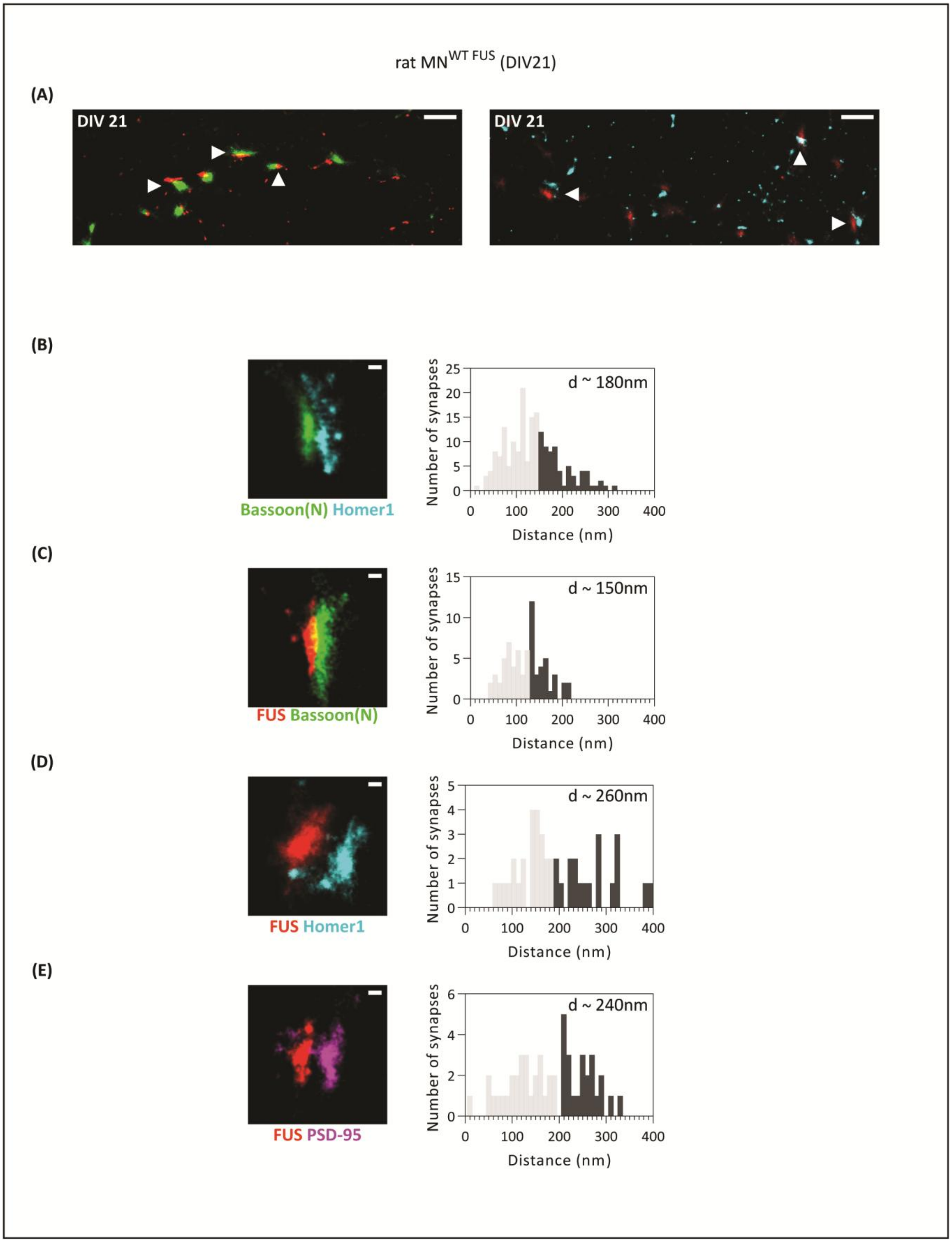
rat MN^WT FUS (DIV21)
(A)
DIV 21
DIV 21
(B)
Bassoon(N) Homer1
d ~ 180nm
Number of synapses
Distance (nm)
(C)
FUS Bassoon(N)
d ~ 150nm
Number of synapses
Distance (nm)
(D)
FUS Homer1
d ~ 260nm
Number of synapses
Distance (nm)
(E)
FUS PSD-95
d ~ 240nm
Number of synapses
Distance (nm)

Supplementary Figure 6: Primary rat MN$^{WT\ FUS}$ at DIV 21 show FUS clustering in the axonal compartment of mature synapses. An overview of mature synapses observed in rat primary motoneurons at DIV 21, labelled for FUS and **(A)** presynaptic protein Bassoon(N), or **(B)** postsynaptic marker Homer1. **(B-E)** Images of single synapses (left) for labelling of- (**B)** Bassoon(N) & Homer1 **(C)** FUS & Bassoon**, (D)** FUS & Homer1 and **(E)** FUS & PSD95. The right panels show histograms of distances between the COM of the respective protein clusters. The lower 60% of the distances are shown in grey. The upper 40% distances are in black and their mean distance 'd' is given. Scale bars are 500 nm for the overview and 100 nm for the single synapses.

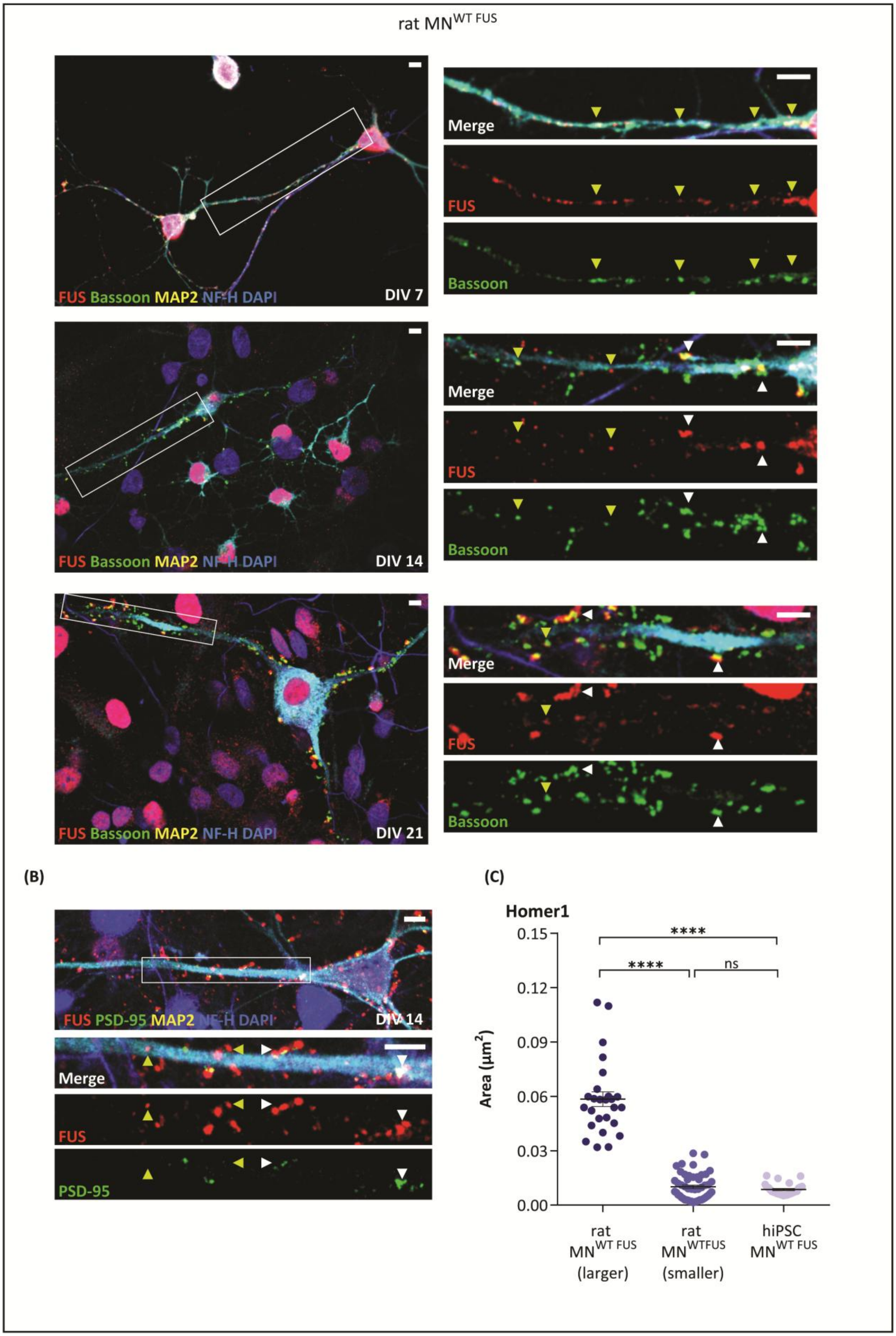
rat MN^WT FUS
Merge
FUS
Bassoon
FUS Bassoon MAP2 NF-H DAPI
DIV 7
DIV 14
DIV 21
(B)
(C)
FUS PSD-95 MAP2 NF-H DAPI
DIV 14
PSD-95
Homer1
Area (µm²)
0.15
0.12
0.09
0.06
0.03
0.00
****
****
ns
rat MN^WT FUS (larger)
rat MN^WTFUS (smaller)
hiPSC MN^WT FUS

Supplementary Figure 7: Synapses show varying protein cluster areas based on the maturation stage. (A-B) Confocal image overview of rat $MN^{WT\ FUS}$ at DIV 7, 14 or 21. The magnified area (white box in the overview) are shown either as merged or single channel images. The white arrowheads mark the bigger synapses present closer to the soma and the yellow arrowheads mark the smaller synapses located at the cell periphery. **(A)** Comparative image of rat $MN^{WT\ FUS}$ at DIV 7, 14 and 21 labelled for Bassoon(N), FUS (red), the dendrite marker MAP2 (yellow), the axonal marker NFH and DAPI (both blue), show an increase in the number of mature synapses with longer culturing time. **(B)** Rat MN cells at DIV 14 show presence of PSD95 (green) in FUS-positive larger synapses but not in the smaller immature synapses. **(C)** The mean and SEM of Homer1 synaptic cluster area, obtained from SMLM imaging data, are shown for mature (> 0.03 μm^2) and developing synapses of rat $MN^{WT\ FUS}$, and for human $MN^{WT\ FUS}$ synapses. Outlier data points were removed. Comparison of data was performed by one way ANOVA ($p < 0.0001$) with Tukey's multiple comparison test., ∗∗∗∗$p \leq 0.0001$. All scale bars are 5 µm.

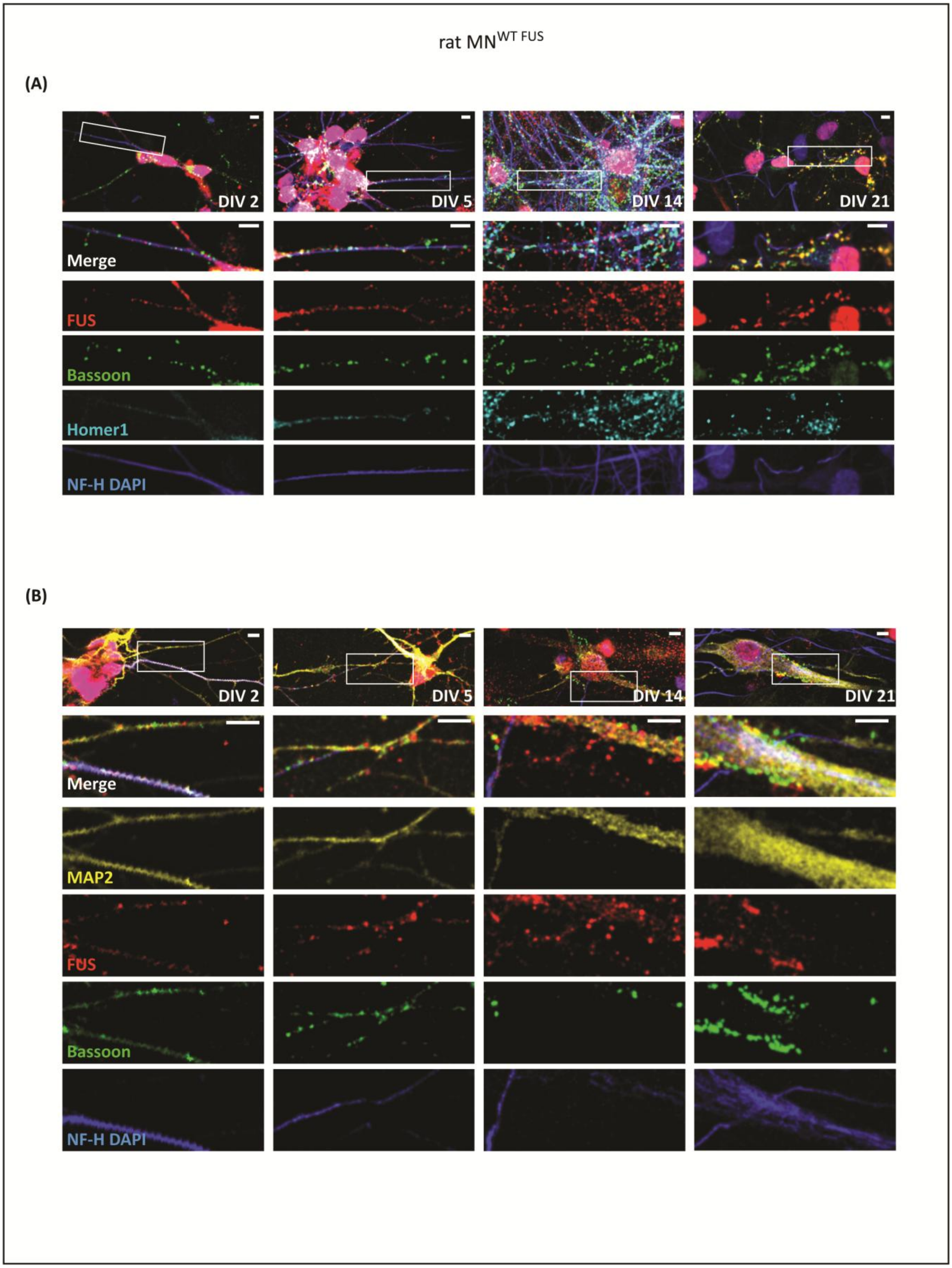
rat MN^WT FUS
(A)
DIV 2
DIV 5
DIV 14
DIV 21
Merge
FUS
Bassoon
Homer1
NF-H DAPI
(B)
DIV 2
DIV 5
DIV 14
DIV 21
Merge
MAP2
FUS
Bassoon
NF-H DAPI

Supplementary Figure 8: In primary rat motoneurons, FUS associates with both axons and dendrites during development. The first row shows an overview confocal image of primary rat $MN^{WT\ FUS}$ at culture stages of DIV 2, 5, 14 and 21 labelled for **(A)** Bassoon(N) (green), FUS (red), Homer1 (cyan), NFH and DAPI (both blue) or **(B)** MAP2 (yellow), FUS (red), Bassoon (green), NFH and DAPI (both blue). The lower rows show a magnified area (white box) as merged or single channel images. All scale bars are 5 µm.
